# Patients with mild paraquat poisoning treated with prolonged low-dose methylprednisolone have better lung function: A retrospective analysis

**DOI:** 10.1097/MD.0000000000010430

**Published:** 2018-04-20

**Authors:** Jie Gao, Zongxun Cao, Shunyi Feng, Yangying Song, Wenjing Bai, Shumin Zhao, Suli Zhang, Yong Li

**Affiliations:** a Emergency Department, Cangzhou Central Hospital, Cangzhou City; b Laboratory Department, Yutian County Hospital, Tangshan City; c Emergency Department, Cangzhou Hospital of Integrated Traditional and Western Medicine, Cangzhou City, China.

**Keywords:** lung function, methylprednisolone, paraquat

## Abstract

Lung dysfunction is an important characteristic of injury induced by paraquat (PQ). This study aimed to evaluate the effects of prolonged low-dose methylprednisolone (MP) treatment on lung function in patients with mild PQ poisoning. We analyzed the results of lung function testing in all patients with mild PQ poisoning admitted to Cangzhou Central Hospital between January 2012 and August 2017. Patients were grouped according to short-term treatment (3 mg/kg/day MP for 3 days) or prolonged treatment (3 mg/kg/day MP for 3 days, followed by dosage reduction by half every 3 days, with treatment terminated when a dosage of 0.375 mg/kg/day was reached). Lung function was evaluated at 2 to 3 months after PQ exposure. The forced expiratory volume in 1 second (85.72 ± 4.93% vs 78.41 ± 4.58%; *P* < .001), forced vital capacity (81.98 ± 4.93% vs 77.85 ± 4.37%; *P* < .001), and diffusing capacity (84.27 ± 5.16% vs 76.21 ± 3.71%; *P* < .001) in the prolonged low-dose MP group were improved compared with those in the short-term MP group. Patients with mild PQ poisoning treated with prolonged low-dose MP had better lung function 2 to 3 months after PQ poisoning.

## 1 Introduction

Paraquat (PQ) is a fast-acting, nonselective bipyridylium herbicide that is widely used in many countries due to its low cost and effectiveness against a wide range of weeds. As the main target organ, the lungs can rapidly develop pulmonary fibrosis associated with respiratory failure, which is the main cause of death in the late stages of PQ poisoning.

Lung fibrosis causes a decrease in inspiratory capacity due to stiffening of the lungs, which reduces ventilatory capacity. In addition, fibrosis also reduces the lung diffusion capacity, leading to hypoxemia. Although prolonged glucocorticoid treatment has provided potential benefit,^[1–5]^ spontaneous improvement has rarely been achieved in PQ ingestion, especially following a sublethal dose. Therefore, we conducted the present study to evaluate the effect of prolonged low-dose methylprednisolone (MP) treatment on lung function in patients with PQ poisoning.

## 2 Methods

### 2.1 Patients

This was a retrospective, observational study based on the results of lung function testing of patients with acute mild PQ poisoning admitted to the emergency department between January 2012 and August 2017. We analyzed the data from patients over 18 years of age who presented to our emergency department within 8 hours of PQ poisoning, with a light blue or barely distinguishable blue color in the urine sample at the time of presentation. Exclusion criteria were incomplete lung function records and the presence of interfering or confounding factors, such as smoking, cardiopulmonary disease, connective tissue disease, pregnancy, or lactation. This study complied with the guidelines of the Declaration of Helsinki, and approval for the study was obtained from the Institutional Review Board. Each patient provided written informed consent for lung function testing during follow-up visits at 2 to 3 months after PQ exposure and for retrospective review of existing data.

### 2.2 Grouping and treatment

According to the therapeutic regimens, all enrolled patients fell into one of the following groups: short-term MP group: (3 mg/kg/day intravenous MP for three consecutive days),^[6]^ and prolonged low-dose MP group: (3 mg/kg/day MP for 3 days, followed by dosage reduction by half every 3 days, with MP treatment terminated when 0.375 mg/kg/day was reached).^[7]^ Lung function was evaluated at 2 to 3 months after PQ exposure.

### 2.3 Data collection

Data extraction was performed by 2 reviewers (Shunyi Feng and Zongxun Cao) independently by adapting a standardized procedure. The primary outcome measures were forced expiratory volume in 1 second (FEV1), forced vital capacity (FVC), and carbon monoxide diffusing capacity (DLco). Any result below 80% of the predicted value was considered indicative of abnormal lung function. We also recorded the age, sex, time from ingestion to arrival, pulse oximetry saturation (SpO_2_) at room air upon arrival, serum creatinine upon arrival, and serum alanine aminotransferase (ALT) upon arrival.

### 2.4 Statistical analysis

All statistical analyses were performed with SPSS (version 13; SPSS Inc., Chicago, IL) for Microsoft Windows. All probabilities were 2-sided, with *P* < .05 considered statistically significant. The results were presented as the means ± standard deviations and were assessed using the independent samples *t* test when the data fit a normal distribution. Otherwise, the results were presented as medians and interquartile ranges, which were assessed using the 2 independent samples and nonparametric tests, respectively. Categorical variables were expressed as percentages and were assessed using the Chi-square test.

## 3 Results

### 3.1 Clinical characteristics of patients with PQ poisoning

The study cohort included 92 patients diagnosed with acute mild PQ poisoning with complete lung function records. Among the 92 enrolled subjects, 34 (37%) cases received short-term MP treatment and 58 (63%) received prolonged low-dose MP treatment. Table 1 summarizes the characteristics of the 2 groups collected upon admission according to the therapeutic regimens of MP and showed no significant differences based on age, sex, time from ingestion to arrival, SpO_2_ on room air upon arrival, serum creatinine upon arrival, and serum ALT upon arrival (all *P* > .05).

**Table 1:**
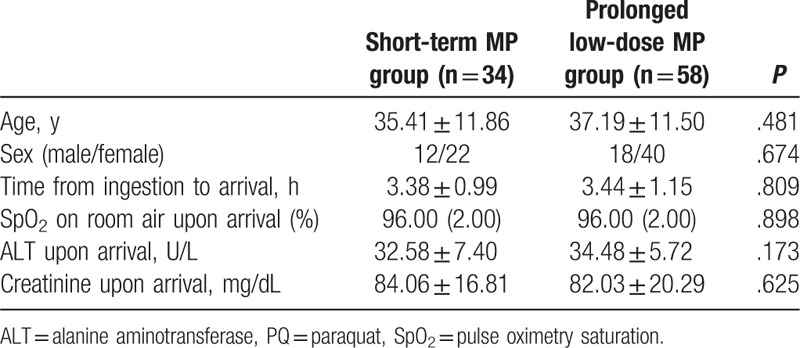
Clinical characteristics of 92 survivors with PQ poisoning.

### 3.2 Main results

The FEV1% (85.72 ± 4.93% vs 78.41 ± 4.58%; *P* < .001), FVC% (81.98 ± 4.93% vs 77.85 ± 4.37%; *P* < .001), and DLco% (84.27 ± 5.16% vs 76.21 ± 3.71%; *P* < .001) values were better in the prolonged low-dose MP group than in the short-term MP group (Table 2).

**Table 2:**
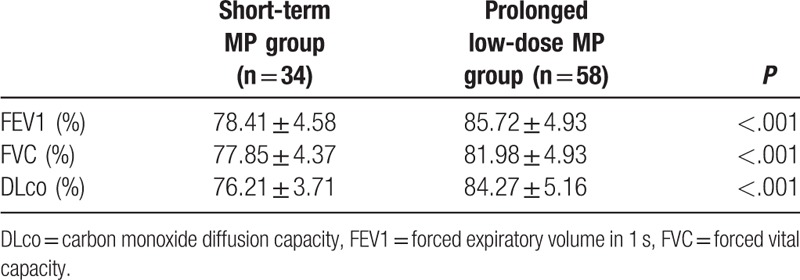
Lung function in 92 survivors of PQ poisoning.

### 3.3 Safety evaluation

Prolonged low-dose MP treatment increased the occurrence of leukopenia (5.9% vs 13.58%; *P* = .239). However, no significant difference was observed. Furthermore, the leukopenia rapidly resolved after MP withdrawal. In addition, no other adverse effects, such as hair loss, acne, infection, or avascular necrosis of the femoral head, were evident from the medical records.

## 4 Discussion

The present study provides further insight into the effects of prolonged low-dose MP treatment on lung function in survivors of mild PQ poisoning. Our results showed that prolonged low-dose MP treatment improves lung function in patients with mild PQ poisoning. In addition, our result showed that the survivors of acute mild PQ poisoning showed less diffusion dysfunction and restriction ventilation dysfunction at 2 to 3 months.

Prolonged glucocorticoid use was chosen because high levels of PQ can be observed in lung samples several weeks after ingestion,^[8]^ and potential damage is consistent with observations from clinical and pathological studies.^[9,10]^ Some studies indicated high efficacy of prolonged glucocorticoid therapy, whereas others showed failure of glucocorticoid therapy. Feng et al^[11]^ reported that prolonged MP treatment after pulse therapy for PQ-intoxicated rats can effectively ameliorate acute lung injury. Subsequently, Gao et al^[7]^ performed a retrospective analysis and demonstrated that prolonged MP therapy after pulse treatment can reduce mortality in patients with moderate-to-severe PQ poisoning. Conversely, Perriëns et al^[12]^ failed to find any significant difference in respiratory failure and survival rates. This difference may have been attributable to the effects of variables, such as PQ dose, glucocorticoid dose, and administration.

Although numerous long-term lung function studies have been performed in survivors of PQ poisoning, the effects of variables, such as PQ dose, exposure duration, and follow-up time, were not completely comparable. Some studies^[13–15]^ reported a decrease in DLco and restrictive effects on FVC and FEV1 after intoxication, consistent with our study findings. In contrast, other studies showed that decreased FEV1%, FVC%, and DLco% values returned to normal after 3 months among survivors of moderate and severe PQ poisoning.^[16]^ The reasons might be explained as follows: patients included in previous reports were younger, with an average age of 28 years; our study patients had more severe PQ-induced lung dysfunction; and the follow-up time was longer than in our study and impaired lung function can recover with time.

This study should be interpreted while considering the following limitations. First, there was possible bias due to a study design that considered only a single point of lung function measurement at 2 to 3 months after PQ poisoning. As we did not have lung function measurements after the initial treatment period or at several points during follow-up, we cannot claim that the results we presented are due to differences in treatment strategies. Thus, we can only state that the group receiving prolonged treatment had better lung function 2 to 3 months after PQ poisoning. Second, our outcomes are based on retrospective data with a small sample size. The positive effect might have been exaggerated and might have resulted in a false-positive result due to small sample size. Thus, we will continue to conduct prospective studies to validate our conclusions. Third, because of the retrospective study design, an element of selection bias may be observed.

In conclusion, patients receiving prolonged treatment had better lung function 2 to 3 months after PQ poisoning. However, well-designed, prospective cohort studies are needed to validate our findings.

## Acknowledgment

The authors would like to thank Li Zhao and Yonghong Liu for assistance in statistical analysis.

## Author contributions

**Conceptualization:** Jie Gao, Zongxun Cao, Shunyi Feng, Yangying Song, Wenjing Bai, Shumin Zhao, Suli Zhang, Yong Li.

**Data curation:** Shumin Zhao.

**Formal analysis:** Jie Gao, Shunyi Feng.

**Investigation:** Wenjing Bai.

**Methodology:** Yangying Song, Suli Zhang.

**Project administration:** Jie Gao, Shunyi Feng, Yong Li.

**Resources:** Yangying Song, Wenjing Bai, Shumin Zhao.

**Software:** Zongxun Cao, Shumin Zhao.

**Supervision:** Shumin Zhao.

**Validation:** Wenjing Bai.

**Writing – original draft:** Zongxun Cao, Shunyi Feng, Yangying Song, Wenjing Bai.

**Writing – review & editing:** Jie Gao, Yong Li.

## References

[1] Yu G Kan B Jian X A case report of acute severe paraquat poisoning and long-term follow-up. Exp Ther Med 2014;8:233–6. 24944627 10.3892/etm.2014.1727 PMC4061211

[2] Descatha A Mégarbane B Garcia V Delayed immunosuppressive treatment in life-threatening paraquat ingestion: a case report. J Med Toxicol 2009;5:76–9. 19415592 10.1007/BF03161092 PMC3550329

[3] Chen GH Lin JL Huang YK Combined methylprednisolone and dexamethasone therapy for paraquat poisoning. Crit Care Med 2002;30:2584–7. 12441774 10.1097/00003246-200211000-00030

[4] Addo E Ramdial S Poon-King T High dosage cyclophosphamide and dexamethasone treatment of paraquat poisoning with 75% survival. West Indian Med J 1984;33:220–6. 6523848

[5] Agarwal R Srinivas R Aggarwal AN Experience with paraquat poisoning in a respiratory intensive care unit in North India. Singapore Med J 2006;47:1033–7. 17139398

[6] Young SL Silbajoris R Dexamethasone increases adult rat lung surfactant lipids. J Appl Physiol (1985) 1986;60:1665–72. 3754860 10.1152/jappl.1986.60.5.1665

[7] Gao J Feng S Wang J Prolonged methylprednisolone therapy after the pulse treatment for patients with moderate-to-severe paraquat poisoning: a retrospective analysis. Medicine (Baltimore) 2017;96:e7244. 28640126 10.1097/MD.0000000000007244 PMC5484234

[8] Licker M Schweizer A Hohn L Single lung transplantation for adult respiratory distress syndrome after paraquat poisoning. Thorax 1998;53:620–1. 9797765 10.1136/thx.53.7.620 PMC1745280

[9] Houzé P Baud FJ Mouy R Toxicokinetics of paraquat in humans. Hum Exp Toxicol 1990;9:5–12. 2328151 10.1177/096032719000900103

[10] Huang CJ Yang MC Ueng SH Subacute pulmonary manifestation in a survivor of severe paraquat intoxication. Am J Med Sci 2005;330:254–6. 16284488 10.1097/00000441-200511000-00011

[11] Feng SY Gao J Wang J Effects of prolonged methylprednisolone treatment after pulse therapy for paraquat-intoxicated rats. Hum Exp Toxicol 2018;37:21–6. 28116923 10.1177/0960327117689909

[12] Perriëns JH Benimadho S Kiauw IL High-dose cyclophosphamide and dexamethasone in paraquat poisoning: a prospective study. Hum Exp Toxicol 1992;11:129–34. 1349219 10.1177/096032719201100212

[13] Cha ES Lee YK Moon EK Paraquat application and respiratory health effects among South Korean farmers. Occup Environ Med 2012;69:398–403. 22213838 10.1136/oemed-2011-100244

[14] Yamashita M Yamashita M Ando Y A long-term follow-up of lung function in survivors of paraquat poisoning. Hum Exp Toxicol 2000;19:99–103. 10773838 10.1191/096032700678815729

[15] Lee KH Gil HW Kim YT Marked recovery from paraquat-induced lung injury during long-term follow-up. Korean J Intern Med 2009;24:95–100. 19543486 10.3904/kjim.2009.24.2.95 PMC2698629

[16] Lin JL Liu L Leu ML Recovery of respiratory function in survivors with paraquat intoxication. Arch Environ Health 1995;50:432–9. 8572721 10.1080/00039896.1995.9935979

